# Childhood Tuberculosis in Household Contacts of Newly Diagnosed TB Patients

**DOI:** 10.1371/journal.pone.0040880

**Published:** 2012-07-31

**Authors:** Srichand Batra, Afsheen Ayaz, Ali Murtaza, Shakil Ahmad, Rumina Hasan, Ruth Pfau

**Affiliations:** 1 Marie Adelaide Leprosy Center, Karachi, Pakistan; 2 Department of Pathology and Microbiology, Aga Khan University, Karachi, Pakistan; Statens Serum Institute, Denmark

## Abstract

**Introduction:**

Childhood tuberculosis (TB), although estimated to account for a major proportion of the global TB disease burden, has a lower public health priority. Reliable research and surveillance data on childhood TB is limited in most regions of the world. This study was conducted to assess the burden of childhood TB among the household contacts of new TB patients in Karachi, Pakistan.

**Methods:**

A retrospective analysis of children (<15 years) who were household contacts of new adult TB patients presenting to Marie Adelaide Leprosy Center (MALC) clinics in Karachi during the period of 2008 to 2010 was conducted.

**Results:**

Of the household children contacts (n = 6613) screened, 317 were suspected and 121(1.8%) diagnosed with TB. These included 89 (73.6%) with pulmonary and 32 (26.4%) with extra-pulmonary disease. Smear positivity rate in pulmonary cases was 32.6%. Mean age of children diagnosed with TB was 11.7 (±2.8) years. Within the child-contacts screened, disease was found to be significantly higher among females (2.3%) in comparison to males (1.2%) (p-value <0.01). The commonest relationship of source cases to diagnosed children was the mother (n = 51, 42.1%). The source case was a female for 66.1% (n = 76) of the children.

**Conclusion:**

A smear positivity rate of 32.6% amongst pulmonary cases suggests their potential to spread disease and emphasizes a need to review the contribution of children in transmission of TB within communities. Greater vulnerability of the female child and considerable role of mother in disease transmission highlights a need to increase focus on females in TB control programs in Pakistan.

## Introduction

Tuberculosis (TB) remains a leading cause of morbidity and mortality in all age groups especially in developing countries [1]. Of the 9 million annual TB cases worldwide, about 1 million (11%) are estimated to be children (<15 years of age) [2], [3], [4]. This figure varies from 3 to 25% in different countries [2]. However these results are based on smear positive cases and hence are likely to underestimate the magnitude of TB in children.

Childhood TB remains a neglected entity due to the fact that majority of the cases are smear negative thus considered to contribute less towards disease spread [5], [6]. Moreover the difficulty in accurately diagnosing pulmonary disease in children remains a significant barrier in addressing the issue of childhood TB [5], [7], [8].

While role of childhood TB in disease transmission may be lower than that of adult disease, childhood TB contributes towards a reservoir from which a significant number of future adult cases arise. Furthermore children are substantially at higher risk of developing severe forms of the disease such as miliary TB and TB meningitis [3]. Childhood TB moreover, usually suggests recent transmission, most commonly from an infectious adult with pulmonary or cavitary disease [9]. To enhance early identification and diagnosis of TB in children routine screening of children in close contact to infectious adult cases is thus recommended [10].

Pakistan ranks 8th on the list of 22 high TB burden countries with an incidence rate of 232/100,000 per year [11], [12]. The DOTS program, implemented in the year 2002 was successful in many aspects; however focus on childhood TB has been minimal. The exact prevalence of TB in children is not known. In accordance with data from the National Tuberculosis Control Program Pakistan (NTP) (2001–2004), 4.4% of all smear positive cases are children under 15 years of age [13]. Given the significant role of adult patients in disseminating disease to children, this analysis was conducted to assess the burden of TB in children among the household contacts of new TB patients in Karachi Pakistan.

## Methods

A retrospective analysis of children (<15 years) who were household contacts of new adult TB patients, presenting to 9 Marie Adelaide Leprosy Center (MALC) field clinics in Karachi between January 1, 2008 to December 31, 2010 was conducted. These clinics are located in 9 of the 18 administrative units of Karachi, where TB treatment is provided free of cost. This community based model is a well recognized program both in Pakistan and internationally and has high credibility in the communities in which it operates. MALC routinely screens all household contacts and offers treatment to those diagnosed with TB.

For this study, household contact was defined as a child (<15 years) living in the same house as the adult patient with TB. Those children who had previously received or were on TB treatment at the time of inclusion were excluded. Detailed history and clinical examination of children in contact with a diagnosed TB patient is routinely carried out as part of MALC's TB program. Initial screening criteria used to identify suspected TB cases included low grade fever and weight loss. Additional criteria were cough for more than 3 weeks for pulmonary TB, and localizing signs/symptoms including palpable lymph nodes, headache/vertigo, backache, and loss of sensation for extrapulmonary TB. Children suspected of having pulmonary TB are encouraged to provide morning sputum for microscopy. Three morning sputum samples are examined for each child. Chest X-ray and tuberculin skin test (TST) is carried out by the clinic staff (treating physician) in those unable to produce sputum and those found to be sputum smear negative on microscopy. TST is performed using five tuberculin units (TU) of tuberculin purified protein derivative (PPD)-S with >5 mm induration as cut off. Extrapulmonary TB is diagnosed on basis of clinical information, TB relevant investigations including fine needle aspiration cytology for lymph node TB, detailed report of pleural effusion for TB of the pleura, skin biopsy for skin TB, TST and MRI/CT scans. Cases of TB are defined in accordance with WHO recommendation. Response to therapy is also monitored.

Data was entered and analyzed in SPSS version 19 (IBM SPSS, Chicago, IL, USA). Descriptive statistics were computed for all variables. Means (± Standard deviation) were calculated for continuous variables while frequencies (%) for categorical variables. Chi square test was used to assess associations.

### Ethics statement

Specific ethical approval for the study and consent from the patients was not needed as the data was taken from patient record maintained by Marie Adelaide Leprosy center (MALC) as part of good clinical practice. At registration MALC advises patients and takes verbal consent that the recorded data may be used anonymously in reports, publications and presentations. For this study the data was used anonymously.

## Results

A total of 1994 adult patients were diagnosed with TB during the study period. Of these 16% (n = 320) had extra-pulmonary TB. Among those with pulmonary TB, 65% (n = 1089) were smear positive.

Household contacts of the 1994 adult TB cases were screened. These included 6613 child contacts (<15 years) of which 2662 (40.25%) were males and 3951 (59.7%) females. Of the total children screened, 317 were suspected on the basis of screening criteria and 121 (1.8%) diagnosed with TB (Table 1).

**Table 1:** Characteristics of children diagnosed with tuberculosis (n = 121).

Characteristics	n	%
**Age of child (years)**		
Mean (±SD)	11.7 (±2.8)	
1–5	6	5
6–10	27	22.3
11–14	88	72.7
**Sex**		
Male	32	26.4
Female	89	73.6
**Type of TB**		
Pulmonary	89	73.6
Extra pulmonary	32	26.4
**Sputum smear** 1		
Negative	45	60.8
Positive (1+)	14	18.9
Positive (2+)	6	8.1
Positive (3+)	9	12.1
**Treatment outcome**		
Treatment completed	88	72.7
Cured	26	21.5
Treatment failure	1	0.8
Died	4	3.3
Out transferred	1	0.8
Defaulted	1	0.8
**Source case relationship to child** *		
Mother	51	42.1
Father	18	14.9
Sister	31	25.6
Brother	27	22.3
Cousin	7	5.8
**No. of source cases**		
One	108	89.2
Two	13	10.8
**Sex of source case** 2 *		
Male	44	38.3
Female	76	66.1

1 For 47 children sputum was not obtained.

2 For 6 children sex of the contact source case was not known.

* Multiple response questions.

Pulmonary TB was diagnosed in 89 (73.6%) cases, of which 29 (32.6%) were sputum smear positive, 43 were diagnosed on the basis of chest X'ray changes and TST positivity, 2 on chest X'ray and clinical grounds while 15 were diagnosed on basis of TST positivity and clinical features. Extrapulmonary TB was diagnosed in 32 (26.4%) children, of which 28 were TST positive. These included 19 with histologically confirmed lymph nodes, 2 with biopsy proven skin TB, 5 diagnosed on the basis of lymphocytic exudative pleural effusion and in 6 cases the diagnosis was made on the basis of MRI or CT scan findings.

Mean age of these diagnosed children was 11.7 (±2.8) years. Majority (72.7%) were between 11–14 years age group. TB was diagnosed in 2.3% (n = 89) of female and 1.2% (n = 32) of male child contacts screened. Disease was found to be significantly higher among female compared to male children screened (p-value <0.01).

The commonest relationship of source cases to diagnosed children was the mother (n = 51, 42.1%) followed by sister (n = 31, 25.6%), brother (n = 27, 22.3%) and father (n = 18, 14.9%). The source case was a female for 66.1% (n = 76) of the children.

Treatment was completed on 88 (72.7%) cases; while an additional 26 (21.5%) were cured. WHO criteria were used for treatment completed and cured [2]. Cured was thus defined as an initial smear-positive TB patient who is sputum smear-negative in the last month of treatment and on at least one previous occasion. There was 1 (0.8%) treatment failure (remained sputum smear-positive at 5 months or later after starting treatment), 4 (3.3%) patients died, 1 (0.8%) was transferred out and 1 (0.8%) defaulted.

## Discussion

The study showed that 1.8% of children in household contacts of adult TB patients also had TB. Consistent with earlier reports, extrapulmonary disease was higher (27.3%) among children as compared to the adults (16%) [3]. In view of the limited sensitivity of sputum smear microscopy in children, the number of childhood pulmonary TB cases being reported however is likely to be an under-estimate. Lack of specificity as well as reader variation in radiological interpretation is a further limitation of this study. Despite these limitations a smear positivity rate of 32.6% amongst children with pulmonary TB suggests that children are likely to be a source of disease spread within this population. The data further suggests that the assumption that children do not contribute significantly to disease transmission needs to be reviewed.

The significantly higher number of girls among children diagnosed with TB is alarming. The link between nutritional deficiency and tuberculosis has long been recognized [14], [15]. It is possible to hypothesize that higher frequency of disease in the females may reflect poor nutritional status of the girl child in this region, making them more vulnerable to the disease [16], [17]. Such an association however needs to be further investigated in the context of childhood tuberculosis in this country. Higher frequency of disease in the girl child moreover is consistent with earlier studies that have also reported a relatively higher frequency of TB in female children [18], [19], [20]. These findings call for further research, to better understand the gender differences identified in this study.

Higher frequency of mothers being reported as the contact of diagnosed TB children is consistent with a number of previous studies [21]. The most likely explanation may be the fact that children are close to mothers and spend more time with them. However, this may also be a consequence of the fact that women tend to be diagnosed and treated late, hence are more likely to transmit disease [22]. Social marginalization of women creates gender disparities in access to health care. Additionally the stigma associated with TB is likely to affect women more than men. Generally speaking women are also more likely to neglect their illness and thus develop advanced disease [23].

In conclusion, our data suggests a need to review the assumption that children do not contribute significantly to TB transmission. Greater vulnerability of the girl child and considerable role of the mother in disease transmission emphasizes a call for increased focus on women and female children in the TB control programs in Pakistan.
